# An Improved Method for the Sensitive Detection of Shiga Toxin 2 in Human Serum

**DOI:** 10.3390/toxins10020059

**Published:** 2018-01-31

**Authors:** Xiaohua He, Gianluigi Ardissino, Stephanie Patfield, Luisa W. Cheng, Christopher J. Silva, Maurizio Brigotti

**Affiliations:** 1 Western Regional Research Center, U.S. Department of Agriculture, Agricultural Research Service, 800 Buchanan Street, Albany, CA 94710, USA; stephanie.patfield@ars.usda.gov (S.P.); luisa.cheng@ars.usda.gov (L.W.C.); christopher.silva@ars.usda.gov (C.J.S.); 2 Center for HUS Control, Prevention and Management, Fondazione IRCCS Ca’Granda Ospedale Maggiore Policlinico, Via Commenda 9, 20122 Milano, Italy; ardissino@italkid.org; 3 Department of Experimental, Diagnostic and Specialty Medicine, University of Bologna, Via San Giacomo 14, 40126 Bologna, Italy

**Keywords:** co-immunoprecipitation, enzyme-linked immunosorbent assay, guanidinium chloride, hemolytic uremic syndrome, human serum amyloid P component, Shiga toxins

## Abstract

Shiga toxins (Stx) released by Stx-producing *E. coli* (STEC) are virulence factors that are most closely associated with hemolytic uremic syndrome (HUS), a life-threatening complication of intestinal infections by STEC. Stx have to enter into the circulatory system before they are delivered to target organs and cause damage. The presence of Stx in sera could be a risk indicator for HUS development. However, the detection of Stx, particularly Stx2, has been difficult due to the presence of Stx2-binding components in human serum. Here, we report new ELISA-based methods for the detection of Stx1 and Stx2 in human serum and the effect of guanidinium chloride on enhancing the sensitivity for the detection of Stx2. The recovery rate for Stx2 was 62% when Stx2-spiked serum samples were treated with guanidinium chloride at a concentration of 200 mM, in contrast to 17% without guanidinium chloride treatment. The effectiveness of guanidinium chloride treatment for the detection of Stx2 in human serum was validated using sera from STEC-infected patients. Coimmunoprecipitation results indicated a specific physical interaction between Stx2 and the human serum amyloid P component (HuSAP) in human serum samples. Our in vitro study demonstrated that the inhibition from HuSAP alone for the detection of Stx2 was only 20%, much less than 69.6% from human serum at Stx2 level 10 ng/mL, suggesting that there may be other factors that bind Stx2 in human serum. This study indicates that treatment of serum samples with guanidinium chloride may be useful for the early and sensitive detection of Stx2 in sera of STEC-infected patients, so preventive measures can be adopted in a timely manner.

## 1. Introduction

Shiga toxin-producing *E. coli* (STEC) has been widely acknowledged as one of the major causative agents of foodborne illness. Shiga toxins (Stx) are the primary virulence factors that lead to hemolytic uremic syndrome (HUS), a clinical composite of thrombocytopenia, hemolytic anemia, and thrombotic microangiopathy that contributes to acute kidney injury, often requiring dialysis, which can progress to acute renal failure and death [1]. Currently, there are no specific treatments available for STEC infection other than supportive care [2]. Use of antibiotics is contraindicated due to the potential induction of Stx release [3,4]. There are two types of Stx produced by STEC, Stx1 and Stx2 [5]. They are distinct genetically and antigenically, but possess similar 3D structures and modes of action. Both Stx1 and Stx2 are composed of one A subunit and five B subunits [6]. The A subunit (32 kDa) contains an enzymatically active domain that cleaves a specific adenine base from the 28S rRNA and thus prevents host cell protein synthesis [7]. The B subunits (7.7 kDa each) bind glycolipids on the surface of host cells and facilitate the internalization of the toxin [8]. Epidemiological studies indicate that Stx2 is associated with a higher risk of developing HUS than Stx1 [9]. Stx2 is also 1000 times more toxic for human renal glomerular endothelia cells than Stx1 [10].

Because of the critical role that Stx plays in the development of HUS, it is important and essential to be able to detect the toxin as early as possible, so preventive measures can be adopted in a timely manner. However, free Stx2 has rarely been detected in the blood of HUS patients [11,12]. Bitzan et al. (1993) reported the presence of a non-immunoglobulin factor in human plasma that binds and neutralizes Stx2, but not Stx1 in vitro [13]. This Stx2-neutralizing activity was later found to be present only in human serum, not in animal sera [14]. In 2001, Kimura et al. identified the Stx2-binding component as a human serum amyloid P component (HuSAP) [15]. Recently, we applied a mass spectrometry-based method to detect Stx1 and Stx2 in human serum and found that the addition of guanidinium chloride (GuCl) substantially improved the sensitivity of detection of Stx2, although 20–70% of the toxins were still lost during the process [16]. 

In this study, we report new enzyme-linked immunosorbent assays (ELISAs) for the detection of Stx1 and Stx2 in human serum and the use of GuCl for enhancing the recovery of Stx2 from human serum and HuSAP. We also investigate the interaction between HuSAP and the Stx2 in serum samples and provide direct evidence of their physical binding using co-immunoprecipitation and Western blot analysis. The new ELISA-based method using GuCl is validated with the accurate detection of Stx2 in sera from STEC-infected patients.

## 2. Results and Discussion

### 2.1. Detection of Stx1 and Stx2 in Stx-Spiked Human Serum by ELISA

**Figure 1:**
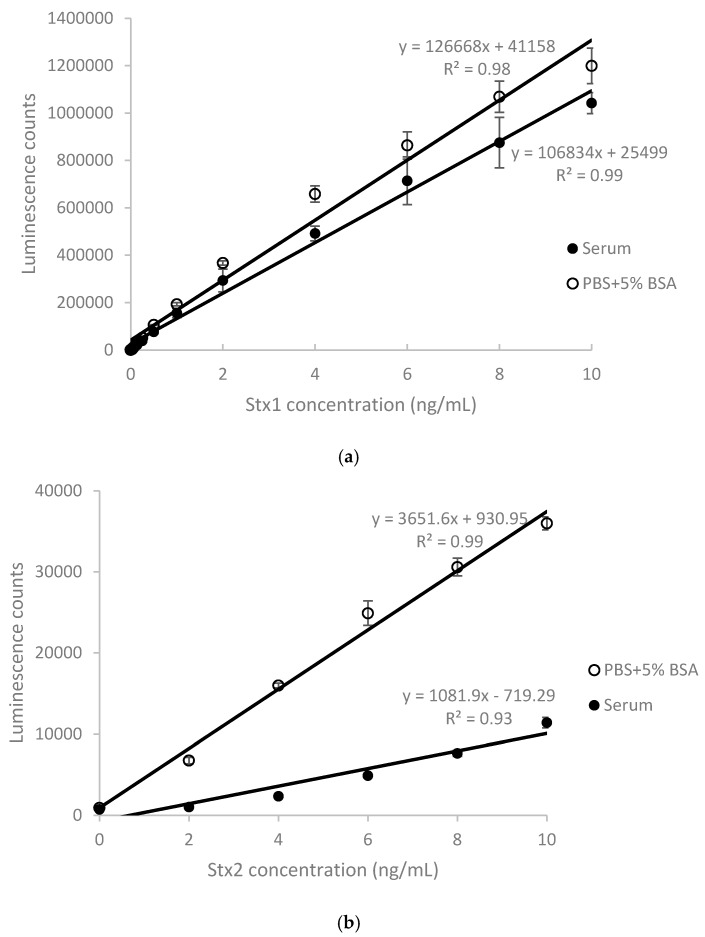
ELISA standard curves performed in PBS and human serum. Data represent the average of the means ± SD (*n* = 6).

Stx plays an essential role in the development of HUS in patients infected by STEC strains. Detection of Stx in the blood of children with STEC-induced HUS [12] suggests that the presence of Stx in serum is a substantial risk factor associated with HUS development. To establish ELISAs that measure Stx in the human blood system, Stx-spiked sera from healthy volunteers were employed. The Stx1 ELISA employed a monoclonal antibody (mAb) against the B-subunit as a capture and another biotinylated B-subunit specific mAb, combined with horseradish peroxidase (HRP)-streptavidin conjugate as detectors. The Stx2 ELISA used a B-subunit specific mAb as a capture, and HRP-conjugated rabbit polyclonal antibody as a detector. In both assays, a chemiluminescent substrate was used for signal development (see Materials and Methods for detailed information). Calibration curves relating the ELISA-based signal to the concentration of Stx1 and Stx2 in phosphate-buffered saline (PBS) supplemented with 5% bovine serum albumin (BSA) and human serum are shown in Figure 1. These curves are linear (R^2^ from 0.93 to 0.99) over a range of 1–10 ng/mL of Stx1 and Stx2 in PBS or serum. The limit of detection (LOD) for Stx1 was 30 pg/mL in both PBS and serum. In contrast, the LOD for Stx2 was 100 pg/mL in PBS and 400 pg/mL in serum. A small matrix effect from human serum was observed with Stx1 detection (Figure 1a). The average recovery rate was 81% within the range of 2 to 10 ng/mL (Table 1). However, a significant matrix effect from human serum was observed with Stx2 detection (Figure 1b), with an average recovery rate of only 17% within the same range.

### 2.2. Effect of GuCl on Detection of Stx2 in Serum

**Figure 2:**
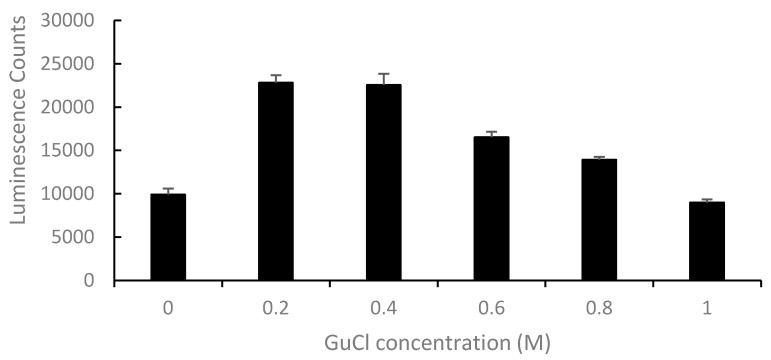
Effect of guanidinium chloride (GuCl) concentrations on the detection of Stx2 in toxin-spiked serum by ELISA.

GuCl is a strong chaotrope and is frequently used to inhibit protein aggregations [17]. Our previous study indicated that there was a large molecule present in human serum that restricted the use of a mass-spectrometry-based method for the detection of Stx2, and the addition of GuCl to the serum significantly enhanced the recovery of Stx2 [16]. It was thought that GuCl may play a role in disrupting the binding between Stx2 and interacting molecules (such as HuSAP) in serum, thereby allowing trypsin access to previously inaccessible Stx2. To examine whether GuCl would improve the ELISA-based detection of Stx2 in human serum, samples were spiked with Stx2 (10 ng/mL) and then treated with different concentrations of GuCl prior to ELISA. ELISA signals increased significantly (*p* ≤ 0.01) when serum samples were pretreated with GuCl at concentrations of 200 and 400 mM (Figure 2). Therefore, 200 mM of GuCl was used to treat serum samples for Stx2 detection.

**Figure 3:**
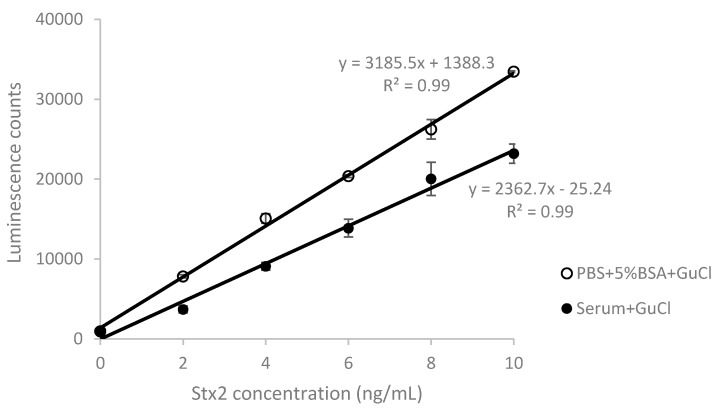
ELISA standard curves performed in PBS and Stx-spiked human serum treated with 200 mM guanidinium chloride (GuCl). Data represent the average of the means ± SD (*n* = 6).

We next tested the recovery of Stx2 in serum treated with 200 mM GuCl (Figure 3). The LOD for Stx2 spiked into human serum samples was lowered to 200 pg/mL and the average recovery rate was increased to 62% within the range of 2 to 10 ng/mL, which is a 3.6-fold increase compared to that obtained from untreated serum samples (Table 2). 

### 2.3. Detection of Stx in Sera of STEC-Infected Patients

To validate the effectiveness of ELISAs developed for this study, serum samples from STEC-infected patients with detectable *stx2* in feces were tested. Stx2 concentrations in serum from patients were estimated using the calibration curves shown in Figure 3. Our results indicated that all serum samples collected from these STEC-infected patients were positive for Stx2. The Stx2 concentrations varied from 0.4 to 4.89 ng/mL (Table 3). The spectrum of clinical manifestations observed in these patients varied from watery or bloody diarrhea to mild renal injury (proteinuria). This is in keeping with the notion that during the intestinal phase of STEC-infections and before the onset of HUS, free Stx are detectable in blood [12,18]. Conversely, free Stx have hardly been detected in the blood of STEC-infected patients after HUS diagnosis [11,12]. It is worth noting that the life-threatening Stx2 was detected in the sera of patients 4 and 5 (Table 3), one day after the onset of STEC-related symptoms, hence at the early stage of STEC infection. Despite the early time points, the amounts of Stx2 recorded in these patients were considerable, i.e., >15-times higher than the LOD. Other fast and accurate methods, such as RT-PCR can demonstrate the presence of *stx* genes in patients at the earliest steps of infection; nevertheless, our improved ELISA directly detects the actual Stx protein molecule in blood mainly responsible for the triggering of the most severe clinical manifestations in STEC-infected patients, such as HUS or bloody diarrhea.

### 2.4. HuSAP Binds Specifically to the Stx2 in Human Serum Samples.

Different approaches have been used to detect free Stx2 in sera [11,12,18], but the percentage of samples with quantifiable serum Stx2 among HUS or STEC-infected patients investigated was very low using standard methods. One proposed explanation was that the Stx2 circulating in blood is bound to HuSAP, which then carries it to target cells [19]. The exact mechanism of Stx2 delivery to its target organs after it gets into the circulation system is still not clear. 

**Figure 4:**
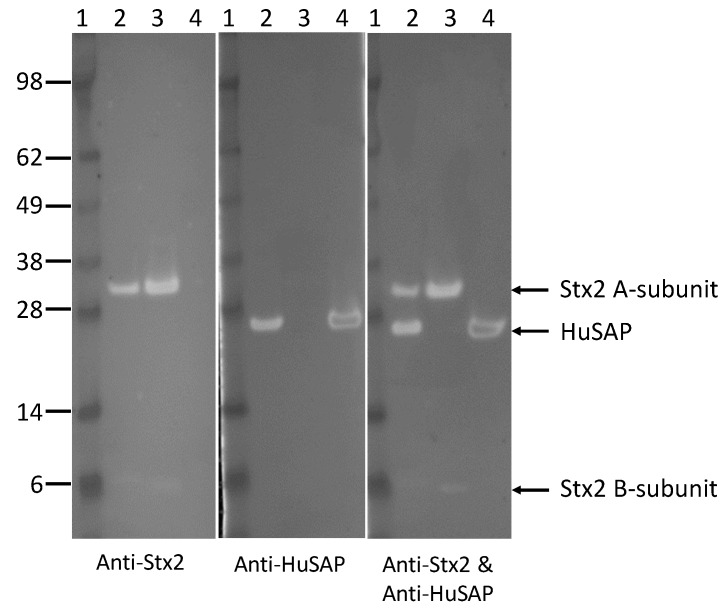
Immunoprecipitation (IP) of Stx2 in human serum. Stx2 (5 µg) was added into 100 µL of human serum and incubated for 30 min at 37 °C, and then subjected to IP with an anti-Stx2 antibody. The pure Stx2, HuSAP, and the immune complex eluted from the affinity column were run on the same SDS-PAGE, and immunoblotted with antibodies against Stx2, HuSAP, or a mixture of antibodies against Stx2 and HuSAP, respectively. Lane 1, Protein markers; lane 2, immunocomplex (10 µL); lane 3, pure Stx2 (0.5 µg); lane 4, pure HuSAP (0.1 µg).

To test if HuSAP interacts with Stx2 in human serum, Stx2 was added into pooled human serum and subjected to immunoprecipitation and then pulled down by passing through a column conjugated with a monoclonal antibody against the B-subunit of Stx2. Stx2 was found to be enriched in the eluted fraction, indicating successful immunoprecipitation. HuSAP was detectable in the eluent when Stx2 was present in the serum (Figure 4), but it was absent when the serum lacked Stx2 (data not shown). These data demonstrate that Stx2 and HuSAP did interact in human serum. 

**Figure 5:**
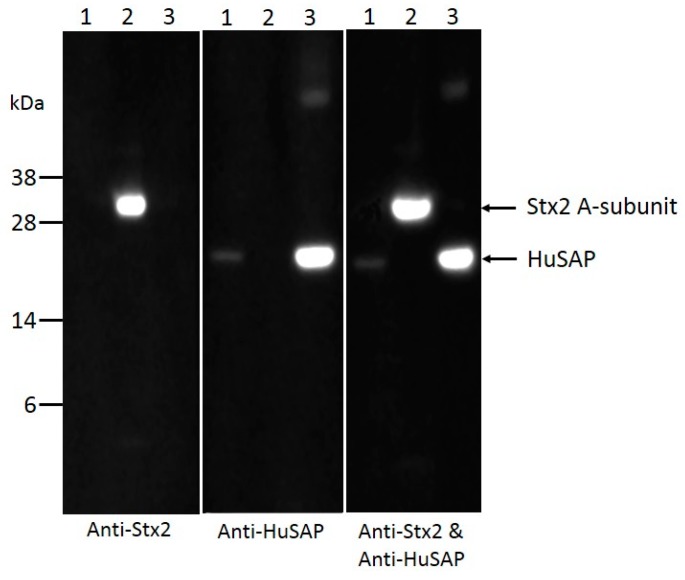
Immunoprecipitation (IP) of Stx2 in serum from an STEC-infected patient. Serum (100 µL) from an STEC-infected patient was subjected to IP with a monoclonal antibody against the Stx2 B-subunit. The pure Stx2, HuSAP, and the immune complex eluted from the affinity column were run on the SDS-PAGE, and immunoblotted with antibodies against Stx2, HuSAP, or a mixture of antibodies against Stx2 and HuSAP, respectively. Lane 1, immunocomplex (10 µL); lane 2, pure Stx2 (0.5 µg); lane 3, pure HuSAP (0.1 µg). Molecular markers are indicated as kilodalton (kDa) at the left side of the panel.

To confirm that Stx2 forms a complex with HuSAP in the sera of STEC-infected patients, similar pull-down experiments were performed by directly passing serum from a STEC-infected patient through the column coupled with a Stx2 antibody. Although Stx2 was not visible, a small amount of HuSAP was found in the immunocomplex pulled down by the Stx2 antibody (Figure 5), suggesting that Stx2 may indeed interact with HuSAP. The failure to detect the Stx2 could be due to the low concentration of Stx2 present in the patient serum and the relatively lower affinity of the Stx2 antibody vs the HuSAP antibody. We normally load 0.5 µg of Stx2 and 0.1 µg of HuSAP in the SDS-PAGE gel to get equivalent signals for Stx2 and HuSAP in Western blots.

**Figure 6:**
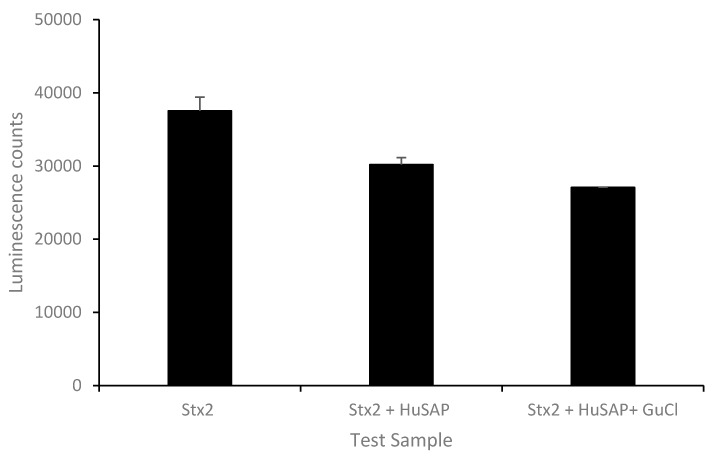
Effect of HuSAP on ELISA detection of Stx2.

To examine if HuSAP is the only factor inhibiting the ELISA-based detection of Stx2 in human serum, commercial HuSAP at 50 µg/mL (HuSAP circulating in human blood ranges from 25 to 50 µg/mL) was mixed with pure Stx2 (10 ng/mL) in the presence of 5% BSA (an analog of human serum albumin) and then tested by ELISA. Figure 6 demonstrates that the ELISA signal obtained from Stx2 samples mixed with HuSAP was significantly lower than that from samples without HuSAP added (*p* ≤ 0.01). However, the inhibition from HuSAP was about 20%, which is much less when compared with the inhibition from the serum spiked with 10 ng/mL of Stx2 (~69.6%), suggesting that in addition to HuSAP, there are likely other components in human serum that interfere with Stx2 detection. Next, we evaluated the ability of GuCl to ablate the inhibition caused by HuSAP. Figure 6 indicates that the average ELISA signal from samples (Stx2 plus HuSAP) treated with GuCl was even lower than samples without GuCl treatment (*p* ≤ 0.05). This result indicates that GuCl is not effective at recovering the inhibition of ELISA-based Stx2 assays from pure HuSAP.

The ELISA inhibition factor in human serum may not be HuSAP or HuSAP may not be the only inhibition factor. Stahl et al. found that Stx2 was present within blood cell-derived microvesicles taken up by renal cortical cells during Shiga toxin-associated infection leading to cell death. This was also observed in Stx2 treated mice and confirmed in in vitro studies, which showed that Stx2-containing blood cell-derived microvesicles undergo endocytosis in glomerular endothelial cells, which leads to cell death [20,21]. Brigotti et al. reported detecting Stx2 in polymorphonuclear leukocytes circulating in the blood of children with HUS [22]. Alternatively, the natural form of HuSAP is different from the isolated form of HuSAP. Hutchinson et al. reported that the HuSAP forms stable pentamers in serum that contains calcium and specific low molecular weight ligands. Isolated HuSAP, however, forms stable decamers in the absence of calcium and low molecular weight ligands, and rapidly auto aggregates in the presence of calcium and absence of low molecular weight ligands [23].

## 3. Conclusions

We have developed ELISAs for the detection of Stx1 and Stx2 in human serum samples. While the recovery of Stx1 in human serum exceeded 80%, it was only 17% for Stx2 using standard ELISAs. To improve the performance of the ELISA for Stx2, human serum samples were treated with GuCl prior to the assay. Recovery of Stx2 increased 3.6-fold using a GuCl concentration of 200 mM. Using this newly improved method, Stx2 was detected in all serum samples collected from STEC-infected patients. Co-immunoprecipitation of Stx2 with human serum confirmed a direct physical binding between Stx2 and HuSAP. Our in vitro results also suggest that HuSAP may not be the only component inhibiting the standard ELISA-based method of detecting Stx2 in human serum. To understand the detailed Stx2-interacting components present in human serum, further studies are needed. Nevertheless, use of this improved ELISA for Stx2 will be helpful to study the kinetics of the toxins before and during the onset of HUS in studies of STEC-infected humans and would help in the early diagnosis of HUS.

## 4. Materials and Methods 

### 4.1. Stx and Antibodies Used

Stx2 was purified from the Stx2a-expressing RM10638 strain as previously described [24]. Stx1 was purchased from Toxin Technologies (Sarasota, FL, USA). Monoclonal antibodies against the B-subunits of Stx1, Stx1-1, and Stx1-2 were produced as previously described [25]. Monoclonal (Stx2-2) and polyclonal (Stx2-pAb) antibodies against Stx2 were prepared as previously described [26,27]. Attachment of HRP to the Stx2-pAb and biotinylation of Stx1-2 was performed using a Lightning-Link HRP Conjugation Kit and Lightning-Link Biotin Conjugation Kit following the manufacturer’s instructions (Innova Biosciences, Cambridge, UK). HRP conjugated streptavidin was purchased from Invitrogen (Carlsbad, CA, USA). HuSAP was purchased from Sigma-Aldrich (St. Louis, MO, USA). Polyclonal antibody against HuSAP (ab27313) was purchased from Abcam (Cambridge, MA, USA).

### 4.2. Human Serum Samples

The human serum samples without Stx contamination were obtained from an FDA licensed commercial donor center and processed in an FDA registered facility (Innovative Research, Inc., Novi, MI, USA). Human serum samples were also collected from patients with a proven STEC-infection on admission to the clinic and frozen in aliquots at −20 °C. Feces specimens were collected as soon as possible after hospitalization. Parents of all patients gave their informed consent for inclusion before they participated in the study. The study was conducted in accordance with the Declaration of Helsinki, and the protocol was approved by the Ethics Committee of the Fondazione IRCCS Ca’ Granda Ospedale Maggiore Policlinico, Milan, Italy (18 May 2010). Detection of *stx* genes in enrichment cultures of feces was performed by PCR-based reverse dot blot or by real-time PCR as previously described [28]. Isolation of STEC strains and the search for antibodies to lipopolysaccharide (LPS) were performed as described in [28].

### 4.3. ELISAs for Detection of Stx1 and Stx2

The Stx1 ELISA entailed the use of 1 µg/mL of capture mAb Stx1-1 to coat the wells, 200 ng/mL of Biotinylated mAb Stx1-2 to detect the presence of captured Stx1, and 125 ng/mL of HRP-streptavidin conjugate. The Stx2 ELISA was performed using 5 µg/mL of capture mAb Stx2-2 (against the B-subunit of Stx2) to coat the wells, and 1 µg/mL of HRP conjugated Stx2-pAb to detect the presence of captured Stx2. In both assays, the plates were coated with capture antibody for 16 h at 4 °C and then blocked with Tris-buffered saline with 0.05% Tween 20, and 3%BSA (for Stx1 ELISA) or 5% nonfat skim milk (for Stx2 ELISA) at 37 °C for 1 h. Sample, detector, and conjugate incubations were all conducted at 37 °C for 1 h. Luminescent count (cps—count per second) was measured after adding 100 µL HRP substrate (SuperSignal Pico) using a Victor 3 plate reader (Perkin-Elmer, Shelton, CT, USA).

Stx standard curves in the serum matrix were prepared by adding various amounts of Stx in 10 µL of PBS into undiluted serum and then diluting the serum 1:10 in PBS. GuCl treatment of serum samples was performed by adding 0.4 M GuCl to an equal volume of serum sample and incubating at 37 °C for 1 h prior to ELISA. The concentrations of Stx in HUS patients were estimated based on the equations from the linear regression of the standard curves. The limit of detection (LOD) was determined using the lowest concentration of analyte that generated a response greater than the background plus three times the standard deviation. All data represent the mean ± SD of three replicates.

### 4.4. Co-Immunoprecipitation Assays

Stx2 (~5 µg) was added to 100 µL of human serum and incubated at 37 °C for 30 min. The mixture was applied to a small column coupled with mAb Stx2-2 and subjected to affinity purification following the manufacturer’s instructions. Protein fractions eluted from the column were tested for the presence of Stx2 and HuSAP by Western blot analysis using specific antibodies against these proteins. 

### 4.5. Polyacrylamide Gel Electrophoresis and Western Blot 

All gel electrophoresis equipment, buffers, gels, and PVDF membranes were purchased from Invitrogen. Proteins were separated by SDS-PAGE using 4–12% NuPAGE (denatured) Novex Bis-Tris mini gels following the manufacturer’s protocol. For Western blot analysis, proteins were electrotransferred to PVDF membranes (0.45 um). The membranes were blocked with 5% nonfat dry milk, and then probed with Stx2 mAb, Stx2-5 [26], at 20 µg/mL, or HuSAP mAb ab27313 (Abcam, Cambridge, MA, USA) at 10 µg/mL, or a mixture of mAbs Stx2-5 and ab27313, followed by goat anti-mouse IgG-HRP at 25 ng/mL (Promega, Madison, WI, USA). Bound antibody was detected using the Amersham ECL-Plus Western Blotting Detection System (GE Healthcare, UK) according to the manufacturer’s protocol. 

## Figures and Tables

**Table 1:** Stx recovery rate from Stx-spiked human serum using ELISA.

Stx Added (ng/mL)	2	4	6	8	10	Mean ± SD
Stx1 recovery (%) ± SD	79.8 ± 11.1	74.6 ± 0.8	82.5 ± 6.2	81.7 ± 4.9	86.9 ± 1.7	81.1 ± 4.9
Stx2 recovery (%) ± SD	4.8 ± 0.8	10.7 ± 0.8	17.4 ± 1.4	23.3 ± 0.6	30.4 ± 0.2	17.3 ± 0.7

**Table 2:** Recovery of Stx2 in toxin-spiked human serum treated with GuCl.

Stx2 Added (ng/mL)	2	4	6	8	10	Mean ± SD
Recovery (%) ± SD	41.23 ± 5.31	58.21 ± 1.04	66.90 ± 5.21	75.72 ± 4.66	68.71 ± 3.53	62.15 ± 3.95

**Table 3:** Clinical characteristics of STEC-infected patients and Stx2 detected in their sera.

Pt.	Age (y)	Sex	Clinical Symptoms	*E. coli* Serotype ^a^	Detection of *stx* Genes in Feces	Stx2 (ng/mL)
1	5.3	F	Diarrhea	nd ^b^	*stx2*+ by PCR-based rev dot blot, RT-PCR	2.99 ± 0.20
2	0.2	M	Bloody diarrhea	O157	*stx2*+ by PCR-based rev dot blot	0.40 ± 0.02
3	0.8	M	diarrhea, proteinuria	O157	*stx2*+ by PCR-based rev dot blot	4.89 ± 0.77
4	5.6	M	diarrhea, proteinuria	O127	*stx2*+ by PCR-based rev dot blot, RT-PCR	3.50 ± 0.75
5	6.3	M	Proteinuria	O127	*stx2*+ by RT-PCR	3.57 ± 0.04

^a^ *E. coli* serotypes in patients 2, 3, and 4 were determined by stool cultures and serotype analysis, in patient 5 by antibody to LPS; ^b^ not determined.

## References

[1] Mayer C.L., Leibowitz C.S., Kurosawa S., Stearns-Kurosawa D.J. (2012). Shiga toxins and the pathophysiology of hemolytic uremic syndrome in humans and animals. Toxins.

[2] Grisaru S. (2014). Management of hemolytic-uremic syndrome in children. Int. J. Nephrol. Renovasc. Dis..

[3] Wong C.S., Mooney J.C., Brandt J.R., Staples A.O., Jelacic S., Boster D.R., Watkins S.L., Tarr P.I. (2012). Risk factors for the hemolytic uremic syndrome in children infected with *Escherichia coli* O157:H7: A multivariable analysis. Clin. Infect. Dis..

[4] Corogeanu D., Willmes R., Wolke M., Plum G., Utermohlen O., Kronke M. (2012). Therapeutic concentrations of antibiotics inhibit Shiga toxin release from enterohemorrhagic *E. coli* O104:H4 from the 2011 german outbreak. BMC Microbiol..

[5] Scheutz F., Teel L.D., Beutin L., Pierard D., Buvens G., Karch H., Mellmann A., Caprioli A., Tozzoli R., Morabito S. (2012). Multicenter evaluation of a sequence-based protocol for subtyping Shiga toxins and standardizing stx nomenclature. J. Clin. Microbiol..

[6] Fraser M.E., Fujinaga M., Cherney M.M., Melton-Celsa A.R., Twiddy E.M., O’Brien A.D., James M.N. (2004). Structure of Shiga toxin type 2 (Stx2) from *Escherichia coli* O157:H7. J. Biol. Chem..

[7] Endo Y., Tsurugi K., Yutsudo T., Takeda Y., Ogasawara T., Igarashi K. (1988). Site of action of a Vero toxin (VT2) from *Escherichia coli* O157:H7 and of Shiga toxin on eukaryotic ribosomes. RNA *N*-glycosidase activity of the toxins. Eur. J. Biochem..

[8] Lingwood C.A. (1993). Verotoxins and their glycolipid receptors. Adv. Lipid Res..

[9] Friedrich A.W., Bielaszewska M., Zhang W.L., Pulz M., Kuczius T., Ammon A., Karch H. (2002). *Escherichia coli* harboring Shiga toxin 2 gene variants: Frequency and association with clinical symptoms. J. Infect. Dis..

[10] Louise C.B., Obrig T.G. (1995). Specific interaction of *Escherichia coli* O157:H7-derived Shiga-like toxin II with human renal endothelial cells. J. Infect. Dis..

[11] Brigotti M., Tazzari P.L., Ravanelli E., Carnicelli D., Rocchi L., Arfilli V., Scavia G., Minelli F., Ricci F., Pagliaro P. (2011). Clinical relevance of Shiga toxin concentrations in the blood of patients with hemolytic uremic syndrome. Pediatr. Infect. Dis. J..

[12] Lopez E.L., Contrini M.M., Glatstein E., Ayala S.G., Santoro R., Ezcurra G., Teplitz E., Matsumoto Y., Sato H., Sakai K. (2012). An epidemiologic surveillance of Shiga-like toxin-producing *Escherichia coli* infection in argentinean children: Risk factors and serum Shiga-like toxin 2 values. Pediatr. Infect. Dis. J..

[13] Bitzan M., Klemt M., Steffens R., Muller-Wiefel D.E. (1993). Differences in verotoxin neutralizing activity of therapeutic immunoglobulins and sera from healthy controls. Infection.

[14] Caprioli A., Luzzi I., Seganti L., Marchetti M., Karmali M., Clarke I., Boyd B. (1994). Frequent and Nature of Verocytotoxin 2 (VT2) Neutralizing Activity (NA) in Human and Animal Sera.

[15] Kimura T., Tani S., Matsumoto Yi Y., Takeda T. (2001). Serum amyloid P component is the Shiga toxin 2-neutralizing factor in human blood. J. Biol. Chem..

[16] Silva C.J., Erickson-Beltran M.L., Skinner C.B., Patfield S.A., He X. (2015). Mass spectrometry-based method of detecting and distinguishing type 1 and type 2 Shiga-like toxins in human serum. Toxins.

[17] Dunbar J., Yennawar H.P., Banerjee S., Luo J., Farber G.K. (1997). The effect of denaturants on protein structure. Protein Sci..

[18] Arfilli V., Carnicelli D., Ardissino G., Torresani E., Scavia G., Brigotti M. (2015). A rapid and sensitive method to measure the functional activity of Shiga toxins in human serum. Toxins.

[19] Kimura T., Tani S., Motoki M., Matsumoto Y. (2003). Role of Shiga toxin 2 (Stx2)-binding protein, human serum amyloid p component (HuSAP), in Shiga toxin-producing *Escherichia coli* infections: Assumption from in vitro and in vivo study using HuSAP and anti-Stx2 humanized monoclonal antibody TMA-15. Biochem. Biophys. Res. Commun..

[20] Stahl A.L., Arvidsson I., Johansson K.E., Chromek M., Rebetz J., Loos S., Kristoffersson A.C., Bekassy Z.D., Morgelin M., Karpman D. (2015). A novel mechanism of bacterial toxin transfer within host blood cell-derived microvesicles. PLoS Pathog..

[21] Villysson A., Tontanahal A., Karpman D. (2017). Microvesicle involvement in Shiga toxin-associated infection. Toxins.

[22] Brigotti M., Caprioli A., Tozzi A.E., Tazzari P.L., Ricci F., Conte R., Carnicelli D., Procaccino M.A., Minelli F., Ferretti A.V. (2006). Shiga toxins present in the gut and in the polymorphonuclear leukocytes circulating in the blood of children with hemolytic-uremic syndrome. J. Clin. Microbiol..

[23] Hutchinson W.L., Hohenester E., Pepys M.B. (2000). Human serum amyloid P component is a single uncomplexed pentamer in whole serum. Mol. Med..

[24] Skinner C., McMahon S., Rasooly R., Carter J.M., He X. (2013). Purification and characterization of Shiga toxin 2f, an immunologically unrelated subtype of shiga toxin 2. PLoS ONE.

[25] Skinner C., Patfield S., Stanker L.H., Fratamico P., He X. (2014). New high-affinity monoclonal antibodies against Shiga toxin 1 facilitate the detection of hybrid Stx1/Stx2 in vivo. PLoS ONE.

[26] He X., McMahon S., Skinner C., Merrill P., Scotcher M.C., Stanker L.H. (2013). Development and characterization of monoclonal antibodies against Shiga toxin 2 and their application for toxin detection in milk. J. Immunol. Methods.

[27] He X., Patfield S., Hnasko R., Rasooly R., Mandrell R.E. (2013). A polyclonal antibody based immunoassay detects seven subtypes of Shiga toxin 2 produced by *Escherichia coli* in human and environmental samples. PLoS ONE.

[28] Caprioli A., Scavia G., Morabito S. (2014). Public health microbiology of Shiga toxin-producing *Escherichia coli*. Microbiol. Spectr..

